# Imaging of abdominal complications of COVID-19 infection

**DOI:** 10.1259/bjro.20200052

**Published:** 2021-07-05

**Authors:** Damiano Caruso, Marta Zerunian, Francesco Pucciarelli, Elena Lucertini, Benedetta Bracci, Tiziano Polidori, Gisella Guido, Michela Polici, Carlotta Rucci, Elsa Iannicelli, Andrea Laghi

**Affiliations:** 1 Radiology section, Department of Surgical and Medical Sciences and Translational Medicine, Sapienza University of Rome - Sant'Andrea University Hospital, Rome, Italy

## Abstract

Coronavirus disease 2019 (COVID-19) is a respiratory syndrome caused by severe acute respiratory syndrome coronavirus 2 (SARS-CoV-2) first described in Wuhan, Hubei Province, China in the last months of 2019 and then declared as a pandemic. Typical symptoms are represented by fever, cough, dyspnea and fatigue, but SARS-CoV-2 infection can also cause gastrointestinal symptoms (vomiting, diarrhoea, abdominal pain, loss of appetite) or be totally asymptomatic. As reported in literature, many patients with COVID-19 pneumonia had a secondary abdominal involvement (bowel, pancreas, gallbladder, spleen, liver, kidneys), confirmed by laboratory tests and also by radiological features. Usually the diagnosis of COVID-19 is suspected and then confirmed by real-time reverse-transcription-polymerase chain reaction (RT-PCR), after the examination of the lung bases of patients, admitted to the emergency department with abdominal symptoms and signs, who underwent abdominal-CT. The aim of this review is to describe the typical and atypical abdominal imaging findings in patients with SARS-CoV-2 infection reported since now in literature.

## Introduction

A novel type of Coronavirus, the severe acute respiratory syndrome Coronavirus 2 (SARS-CoV-2) was identified in Wuhan, a city in the Hubei province of China, on December 2019. The associated disease is typically characterized by respiratory symptoms and it was called Coronavirus disease 2019 (COVID-19). The WHO declared pandemic on 11 March 2020.^1^ Real-time reverse-transcription-polymerase chain reaction (RT-PCR) applied on respiratory tract specimens represents the reference standard for the detection of SARS-CoV-2 infection.^2^ Imaging plays an important role on the diagnostic process of the disease^3^ : as shown by Ai T. and colleagues^4^ and Caruso D. and colleagues,^5^ chest CT has a sensitivity of 97%. Typical chest CT findings are bilateral posterior ground-glass opacities (GGOs) and thickening of interlobar and interlobular septa (crazy paving pattern).^6,7^ The first clinical manifestations reported during the spreading were referred to the respiratory tract, with typical symptoms as cough, dyspnea and fever. However, during the pandemic, also other organs seem to be involved in the disease due to systemic effects of the SARS-CoV-2.

Since new information about COVID-19 are released daily, in some cases COVID-19 infection can present primarily with abdominal symptoms, such as abdominal pain, diarrhoea or vomiting^8^ and also hepato-biliary tract injury of uncertain origin have been described in some patients with COVID-19.^9^ Gastrointestinal and abdominal viscera involvement seem to be related to angiotensin converting enzyme 2 (ACE2) expression in the gastrointestinal tract and, although less represented, also in biliary epithelium.^10^ Renal disfunction and pancreatic involvement are more rare, but they were also described in some cases.^11–13^

Thus, the purpose of this review is to describe the typical and atypical abdominal imaging findings of COVID-19.

### Small and large bowel

Gastrointestinal involvement in COVID-19 is a very common extra thoracic manifestation and, in some cases, it could be the only manifestation or the first one, preceding pulmonary involvement. A possible explanation could be the high expression of ACE2 receptors, which are considered the main virus cellular carriers in type II alveolar cells, also on enterocytes membrane.^10^

**Figure 1.**
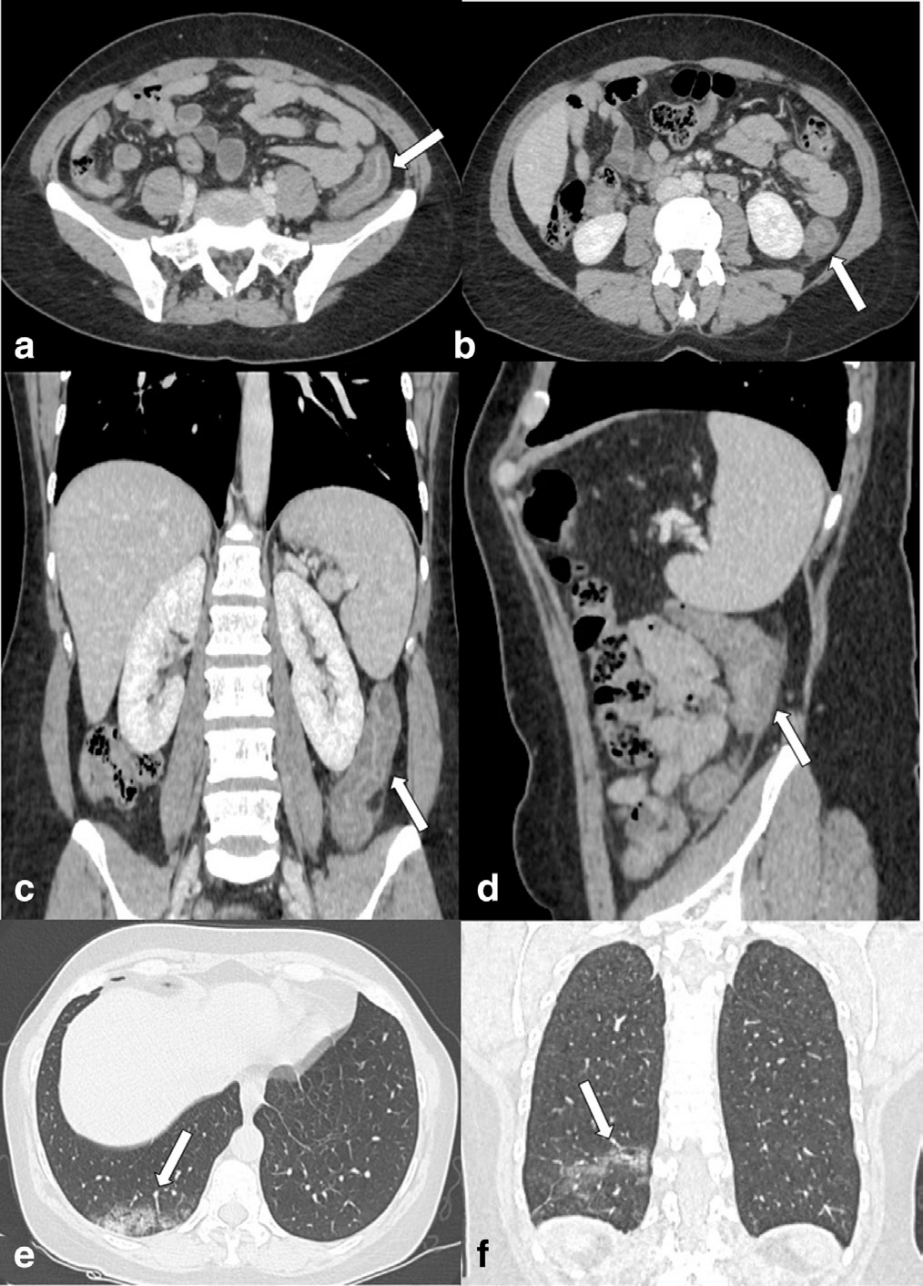
Enhanced CT scan (portal phase) of the abdomen of a 55-yo female admitted to emergency department with abdominal pain and rectorrhagia. (a and b) Axial images, (c) Coronal MPR image and (d) Sagittal MPR image. Principal finding is the wall thickening of the descending colon with enhancing mucosa and muscularis propria with the oedematous submucosa in between (arrows). (e) Axial image, (f) Coronal MPR image. Analysis of lung basis shows the ground-glass opacities (GGOs) of lung parenchyma in the right lower lobe posterior segment suggestive for COVID-19 infection (arrows).

**Figure 2.**
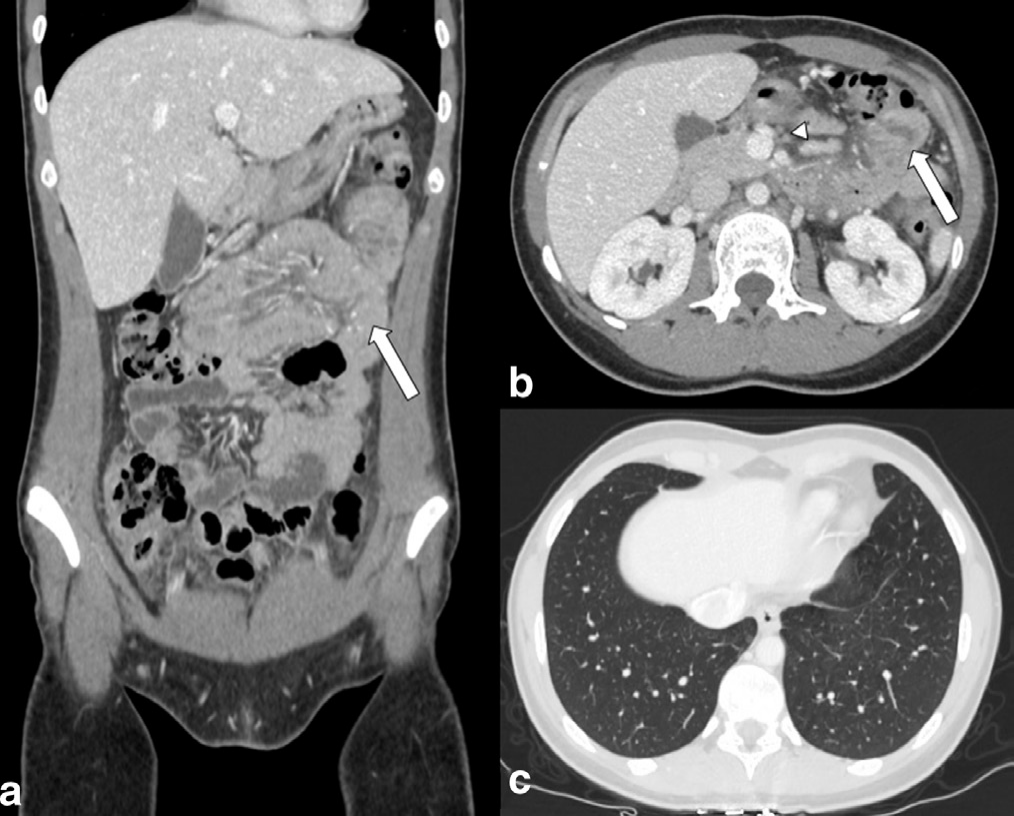
Enhanced CT scan (portal phase) of the abdomen of a 25-yo female admitted to emergency department with abdominal pain and fever. (a) Coronal MPR image, (b) Axial images. Principal finding is the wall thickening and enhancing of the small intestine (arrows). Mesenteric lymphadenopathy is present (arrowhead). (c) Axial image: analysis of lung basis did not show findings suggestive for COVID-19 infection, further confirmed by real-time reverse-transcription polymerase chain reaction (RT-PCR).

Contrast-enhanced CT (CECT) is the modality of choice for the detection of bowel involvement, but also ultrasonography (US) and magnetic resonance (MR) can be useful for management and follow-up. Most typical imaging findings on CT include bowel wall thickening, low-density ring of submucosal oedema between enhancing mucosa and serosa (target sign) (Figures 1 and 2), bowel dilation, pericolic fluid or fat stranding. Locoregional inflammatory nodes can also be detected^14^ (Figure 2).

Pneumatosis Intestinalis (PI) is another possible finding reported in COVID-19.^15^ PI, or intramural bowel gas, related to the presence of gas within the wall of the bowel, is a rare condition with a wide range of clinical manifestations: it could be asymptomatic or present as life-threatening form.^16^ PI can be a primary condition (idiopathic) or, more frequently, a secondary manifestation of several pathological phenomena, such as chronic bowel ischemia, obstructive and necrotic GI diseases, systemic autoimmune diseases and iatrogenic causes.^17^

Small bowel wall inflammation and ischemia due to mesenteric and portal vein thrombosis are typical imaging findings described in COVID-19.^18^

Carvalho et al.^19^ reported a COVID-19 patient presenting abdominal pain and dissention and no respiratory symptoms. Intravenous contrast-enhanced CT scan of the abdomen and pelvis showed severe inflammation of the ascending colon, transverse colon, and descending colon, characterized by circumferential wall thickening, mural hyperenhancement, mesenteric hypervascularity and pericolic fat stranding.

Zhang et al.,^20^ Pan et al.^21^ and Hormati et al.^22^ reported several cases of gastrointestinal complications in COVID-19 patients (39% of patients described by Zhang^20^) and in some cases, abdominal symptoms can occur also before the respiratory manifestations of the disease.^22^

As part of the bowel, the appendix can also be subject to inflammation due to COVID-19 infection; however, nowadays, the literature is limited to case reports. Pautrat et al.^23^ reported a case of suspicious clinical presentation of appendicitis with no respiratory symptoms suspicious for COVID-19 pneumonia. Abdominal CT did not show any typical findings of appendicitis and CT scan of lung bases showed typical imaging findings of COVID-19 interstitial pneumonia confirmed then by RT-PCR. A similar case of clinical diagnosis of appendicitis due to abdominal pain in right iliac fossa, without respiratory symptoms, was reported by Abdalhadi et al^24^. CT was performed and appendicitis diagnosis was excluded, but bilateral patchy peripheral consolidations and GGOs suspicious for COVID-19 were found on lung bases, then confirmed by RT-PCR.

### Liver

Liver injury in COVID-19 could be related to a direct cytopathic effect of the virus in the liver.^25^ In fact, elevated levels of liver enzymes like ALS, AST, and GGT can be found in many SARS-CoV-2 positive patients’ blood. Lenti et al.^25^ reported in their retrospective observational study performed on 100 COVID-19 patients that the 62.4% of patients had liver function test alterations. Xu et al.^26^ documented a moderate micro-vesicular steatosis and mild lobular and portal activity in liver biopsy specimens of COVID-19 patients, that could be related to SARS-CoV-2 infection or drug-induced liver injury. Up to now, no hepatic imaging findings related to COVID-19 have been reported in medical literature; however, patients with SARS-CoV-2 infection with associated laboratory confirmed liver injury can show on CT and ultrasound imaging features of hepatic steatosis.^11^ In addition, some cases of severe acute liver failure in COVID-19 patients were reported in literature.^27^

### Kidneys

The presence of ACE2 receptors on kidneys podocytes and proximal convoluted tubules cells could explain the role of SARS-CoV-2 infection in the pathophysiology of acute renal failure, due to a direct virus-induced cytopathic effect as one of the possible fatal complications of COVID-19.^11^ Zaim et al.^28^ showed that 0.5% up to 19% of patients with COVID-19 have acute renal dysfunction, higher than general population. Thus, may be of relevant information to take extremely care of renal function before to administrate contrast agents for CT and MRI studies in COVID-19 patients. Since COVID-19 is a new disease, imaging still not have a strong correlation with these laboratory findings and no data were reported in literature yet; ultrasound and scintigraphy features of acute renal failure (such as increased parenchymal echogenicity) can be expected in patients with SARS-CoV-2 associated renal injury.^11^

### Pancreas

**Figure 3.**
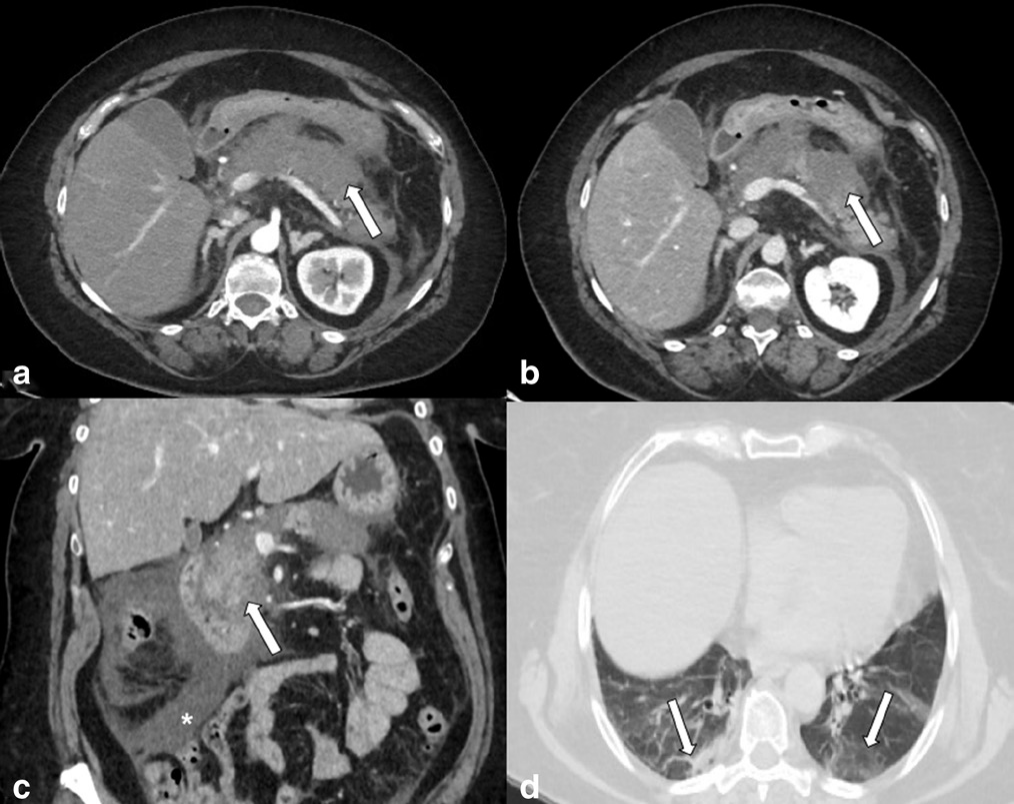
Enhanced CT scan. (a) Arterial phase, Axial image, (b) Portal phase, Axial image (c) Portal phase, Coronal MPR image of the abdomen of a 69-yo female admitted to emergency department with abdominal pain. Principal finding is the enlargement of the pancreas that appears oedematous, with indistinct margins (arrows). A huge amount of fluid is present (asterisk). (d) Axial image: lung basis analysis shows the ground-glass opacities (GGOs) and consolidation of lung parenchyma in both lower lobes posterior segment suggestive for COVID-19 infection (arrows).

**Figure 4.**
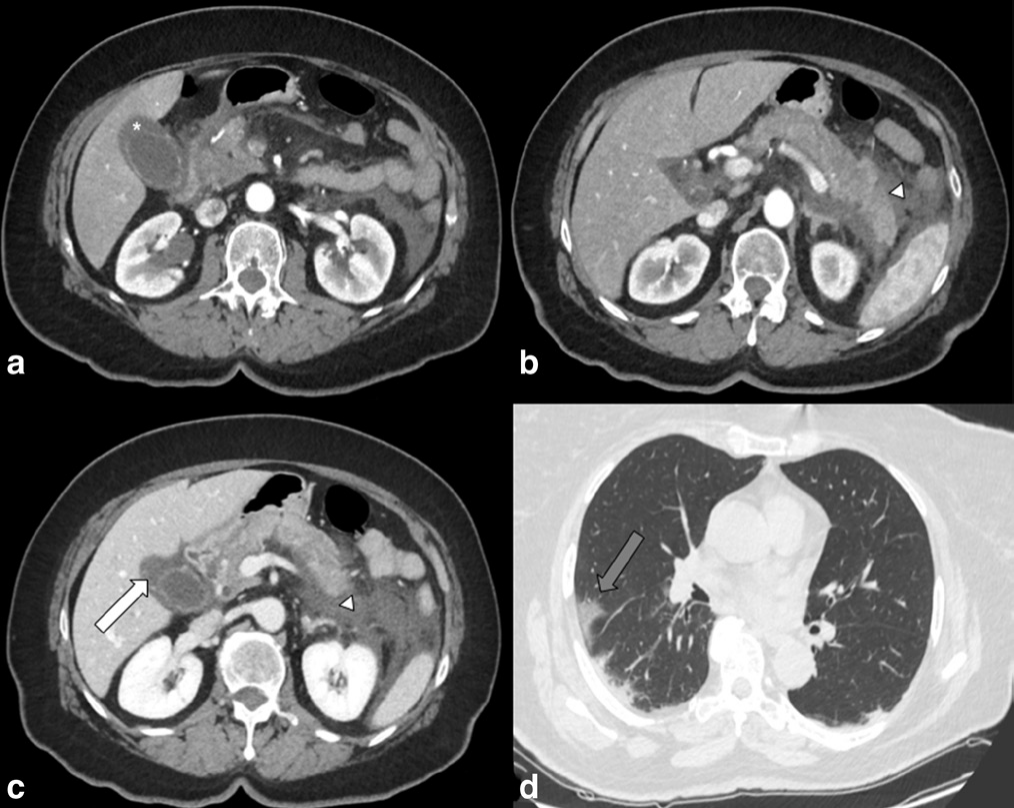
Enhanced CT scan. (a and b) Arterial phase, Axial images, (c) Portal phase, Axial image, of the abdomen of a 62-yo female admitted to emergency department with abdominal pain and fever. Principal findings are the gallbladder wall that appears thickened and hyper enhanced (asterisk) with pericholecystic fluid (white arrow); (b) image shows concomitant diffuse pancreatic parenchymal enlargement, surrounding retroperitoneal fat stranding and fluid collection (arrowhead). (d) Axial image: analysis of lung basis shows the ground-glass opacities (GGOs) of lung parenchyma in the right lower lobe suggestive for COVID-19 infection (grey arrow).

Pancreas damage have been shown in some patient with COVID-19 and abdominal pain, but causes of pancreatic involvement in COVID-19 are not totally clear yet.^29^ Acute pancreatitis is the expression of active inflammation of pancreatic parenchyma. CECT is highly recommended for diagnosis showing a focal or diffuse parenchymal enlargement, changes in density because of oedema, indistinct pancreatic margins owing to inflammation (Figures 3 and 4), surrounding retroperitoneal fat stranding. Typical complication of acute pancreatitis can be peripancreatic fluid collection, pseudocyst, walled-off necrosis defined as an area of lack enhancing and pancreatic abscess.^30^

Schepis et al.^12^ reported the presence of SARS-CoV-2 in the analysis of pancreatic pseudocyst hypothesizing its potential role in the pathogenesis of pancreatic diseases in COVID-19 patient. Hadi et al.^13^ reported a case of acute pancreatitis associated with COVID-19 in 2/3 family members with increasing pancreatic enzymes. Other causes of acute pancreatitis were excluded for both patients (including alcohol, biliary obstruction/gall stones, drugs, trauma, hypertriglyceridemia, hypercalcemia, and hypotension).

Due to the frequent pancreatic involvement on COVID-19 disease, 17% reported by Wang^31^ and 16% by Mukherjee,^29^ it is important to consider pancreatic involvement in COVID-19 for clinical physicians and radiologists. In addition, it is important to carefully evaluate the pancreas in the lowest slices of a chest-CT, as it is often included.

### Gallbladder

**Figure 5.**
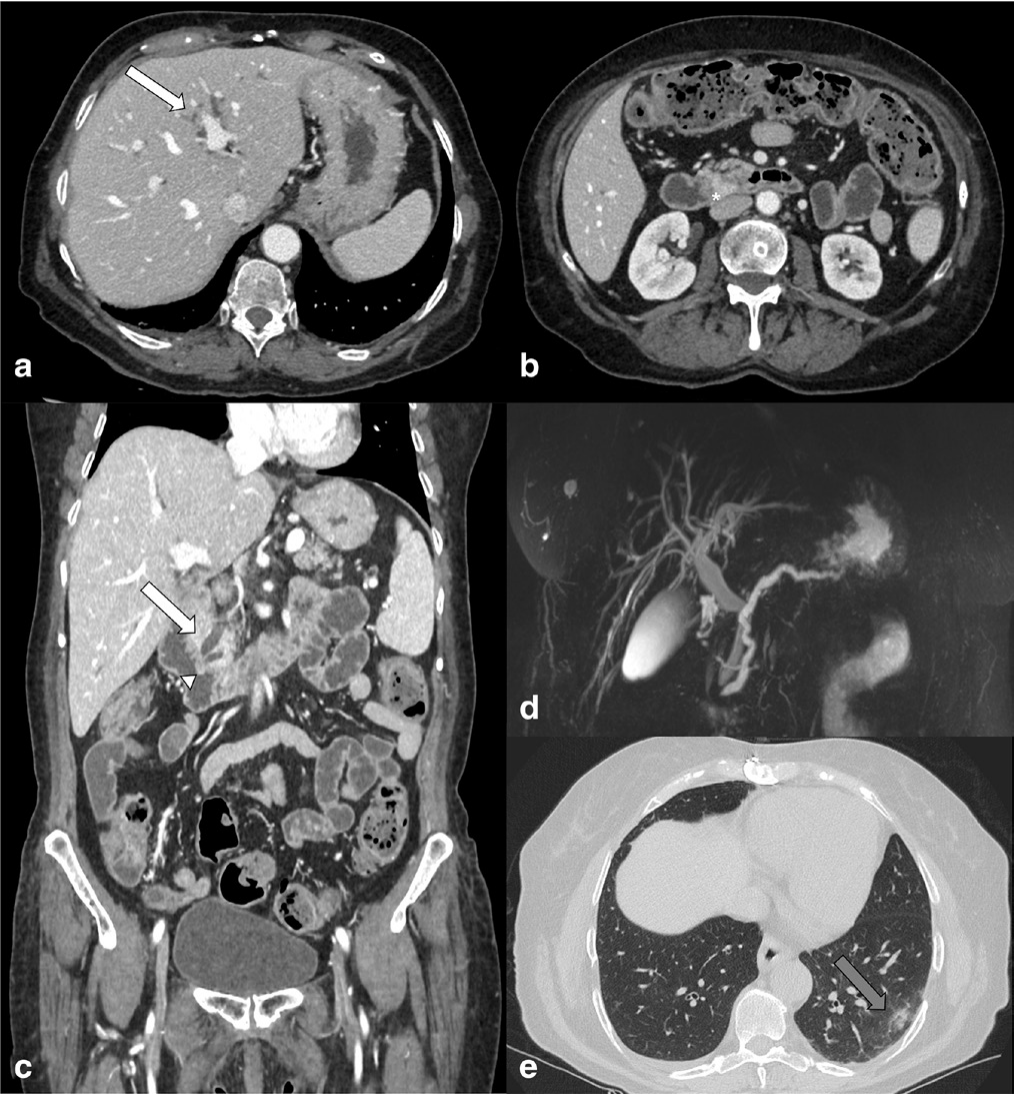
Enhanced CT scan (portal phase) of the abdomen of a 67-yo female admitted to emergency department with abdominal pain, fever and jaundice. (a and b) Axial images, (c) Coronal MPR image. Principal finding is the intrahepatic and extrahepatic biliary dilatation (white arrows) and pancreatic duct dilatation (arrowhead). Enhancement of pancreatic papilla, suggestive for phlogosis, is present (asterisk). (d) Magnetic resonance cholangiopancreatography (MRCP) image shows absence of intraluminal obstruction of biliary system. (e) Axial image: analysis of lung basis shows the ground-glass opacities (GGOs) of lung parenchyma in the left lower lobe suggestive for COVID-19 infection (grey arrow).

Acute cholecystitis is the inflammation of the gallbladder. This condition has been reported in some COVID-19 patients, probably due to the presence of ACE2 receptors in the biliary system (Figure 5). Nevertheless, the virus in bile samples of the studies mentioned below was not found, so this pathogenesis cannot be certainly demonstrated.^10^

CECT is highly sensitive for diagnosis.^32^ Imaging findings include: gallbladder distension, gallbladder wall thickening, mural or mucosal hyperenhancement in post-contrast phases, pericholecystic fluid and inflammatory fat stranding (Figure 4), enhancement of the adjacent liver parenchyma due to reactive hyperemia. Complications include: gangrenous cholecystitis, gallbladder perforation, fistula (cholecystoenteric or cholecystocutaneous) and vascular complications such as portal vein thrombosis and cystic artery pseudoaneurysm.^32^

Ying et al.^33^ reported a case that may be related with COVID-19 in a patient who had constant pain in the right upper quadrant of the abdomen during the hospitalization that was finally diagnosed as acute cholecystitis.^10^

A case of ischemic gangrenous cholecystitis as a tardive complication of COVID-19 is also reported by Bruni A. and colleagues^34^ in a female with acute respiratory failure. This complication could be related by a dysregulated host inflammatory response and thrombosis of medium-size vessels.

### Spleen

Splenic infarction (SI) occurs when splenic blood supply is compromised.^35^ Many cases of SI related to COVID-19 are reported in literature.^36,37^ Sometimes, thrombotic events in COVID-19 can be the initial manifestations of the disease.^36^ Authors underline this aspect due to the prothrombotic effect of COVID-19.^38^ Mild thrombocytopenia^39^ and increased D-dimer levels^40^ are the most consistent hemostatic abnormalities that cause some forms of coagulopathy that may predispose to thrombotic events.

As in non-COVID-19-related SI, appearance of infarction is related to the time of the event. CECT is considered the imaging investigation of choice; typical imaging finding is peripheral, wedge-shaped hypoenhancing region, with normal intervening enhancing splenic tissue.^35^

The incidence of SI is probably an unrecognized diagnosis (due to the few data reported in literature) and radiologists should consider this rare manifestation in the evaluation of chest CT as a collateral finding in the upper abdominal slices.

## Conclusions

In conclusion, abdominal involvement in COVID-19 is not so rare and it should not be underestimated.

The abdominal involvement in COVID-19 can be related in many cases to the ACE2 receptors expression in the epithelium of some abdominal districts or in the vascular tropism that can produce thrombotic events.

As it is not such a rare event, it is important to keep in mind a possible abdominal involvement of COVID-19 in patients who refer general gastrointestinal symptoms. Imaging is a powerful method to detect and follow-up abdominal COVID-19 manifestations and possible related complications to assess the best patients’ management.
